# Preclinical Study of Immunological Isoxazole Derivatives as a Potential Support for Melanoma Chemotherapy

**DOI:** 10.3390/ijms222010920

**Published:** 2021-10-10

**Authors:** Izabela Jęśkowiak, Benita Wiatrak, Adam Szeląg, Marcin Mączyński

**Affiliations:** 1 Department of Pharmacology, Faculty of Medicine, Wroclaw Medical University, Mikulicza-Radeckiego 2, 50-345 Wrocław, Poland; benita.wiatrak@umed.wroc.pl (B.W.); adam.szelag@umed.wroc.pl (A.S.); 2 Department of Organic Chemistry, Faculty of Pharmacy, Wroclaw Medical University, 211A Borowska Str., 50-556 Wrocław, Poland; marcin.maczynski@umed.wroc.pl

**Keywords:** skin cancer, skin diseases, isoxazole derivatives, melanoma

## Abstract

(1) Background: Melanoma is an aggressive neoplasm derived from melanocyte precursors with a high metastatic potential. Responses to chemotherapy and immunotherapy for melanoma remain weak, underlining the urgent need to develop new therapeutic strategies for the treatment of melanoma. (2) Methods: The viability of NHDF and A375 cell cultures after the administration of the tested isoxazole derivatives was assessed after 24-h and 48-h incubation periods with the test compounds in the MTT test. ROS and NO scavenging analyses, a glycoprotein-P activity analysis, a migration assay, a test of apoptosis, and a multiple-criteria decision analysis were also performed. (3) Results: All compounds that were tested resulted in a slower migration of melanoma neoplastic cells. The mechanism of the antitumor activity of the tested compounds was confirmed—i.e., the pro-apoptotic activity of the compounds in A375 cell cultures. Compound O7K qualified for further research. (4) Conclusions: All the tested compounds inhibited the formation of melanoma metastases and demonstrated the ability to reduce the risk of developing drug resistance in the tumor. The MCDA results showed that O7K showed the strongest antitumor activity.

## 1. Introduction

Despite our in-depth knowledge of the pathogenesis of melanoma, prognosis and patient survival remain a challenge. It is estimated that melanoma is responsible for 65%–75% of skin cancer deaths each year [1]. Although the mean age of diagnosis is 63 years, the incidence increases with age. Melanoma affects all demographic groups and is often diagnosed in people under the age of 30. Women generally have a better prognosis than men, possibly due to the interaction of sex hormones with melanoma cells [2]. Other risk factors for developing melanoma include the number of moles, age, UV exposure, and having a family history of the disease. About 30%–50% of all melanomas are derived from moles and are associated with non-chronic sun damage [3].

Melanoma is the most aggressive and dangerous form of skin cancer that develops as a result of melanocyte cancer. The skin consists of two main layers: the epidermis, the outer layer that consists mainly of keratinocytes, and the dermis, which contains fibroblasts, mast cells, macrophages, and Langerhans cells. Melanocytes are located along the basal layer of the epidermis. Their main function is to deliver melanin to the keratinocytes, which leads to the pigmentation of the skin and its protection against damage by ultraviolet (UV) radiation [3]. Moreover, melanoma cells exhibit so-called vascular mimicry, which consists of adopting endothelial features and inducing the expression of pro-angiogenic receptors and ligands [4]. Melanoma preferentially metastasizes through the lymphatic pathway, but can spread through the bloodstream in some cases. The main organs where melanoma metastases can develop are the lungs, brain, liver, and bones [5].

The most effective treatment for melanoma is the surgical resection of the primary tumor and metastases. Nevertheless, systemic treatment is widely used. Unfortunately, its effectiveness is relatively low, with a high toxicity and the rapidly developing resistance to administered drugs. For years, dacarbazine (DTIC) has been a key drug in the treatment of advanced melanoma [6]. Immunotherapeutic agents such as interleukin 2 (IL-2) and interferon a (IFN-α) have also been used in therapy [7]. However, none of these agents have been shown to significantly extend the overall survival of patients, and their use is associated with serious side effects. Over the past few years, the prognosis of patients with advanced melanoma has been improved by the use of programmed cell death antibody 1 (PD-1), anti-cytotoxic antibody protein 4 associated with T-lymphocytes (CTLA-4), BRAF inhibitors, and MEK inhibitors [8]. In order to minimize the side effects of systemic treatment and increase the effectiveness of chemotherapy, the best solution seems to be the development of a therapy directed against molecules specific to each type of cancer [7]. Unfortunately, melanoma is one of the most immunogenic neoplasms and is related to the formation of a large number of neo-antigens as a result of chromosomal rearrangements or genetic polymorphisms. Therefore, it is difficult to design an appropriate onco-logical therapy when immune cells are the target of modern antimelanoma therapy. Unfortunately, as with BRAF and MEK inhibitors, people with melanoma sometimes do not respond to therapy or become resistant to this form of treatment [6]. 

In order to find a suitable drug substance for use against melanoma, isoxazole derivatives with immunomodulatory activity were selected and tested for their antitumor activity. The compounds O2K (M2), O3K (M3), O4K (M7), and O5K (M8) were selected for the tests from the M series, also known as OK series, as demonstrated in Table 1. Isoxazole derivatives are characterized by a broad panel of activities, such as anticancer, antibacterial, antiviral, immunomodulating, anti-inflammatory, analgesic, and anticonvulsant [9]. The immunoregulatory properties of isoxazole derivatives can be classified into several categories, such as immunosuppressive, anti-inflammatory, and immunostimulatory, the effects of which have been studied in cell cultures and animals [10].

Interesting activity was also shown by coumarin derivatives containing isoxazole groups, which showed a better activity in the synthesis of melanin than 8-methoxypsolar and thus were considered the most promising candidates for further pharmacological studies on vitiligo [11]. Similar results were found for the isoxazole-chalcone derivative PMPP. It has been shown that this compound can effectively induce melanogenesis in B16 cells by increasing GSK3β phosphorylation through Akt activation [12]. Moreover, isoxazole derivatives show a low toxicity and good bioactivity at low doses [10]. 

## 2. Results

### 2.1. Viability Assay

**Figure 1:**
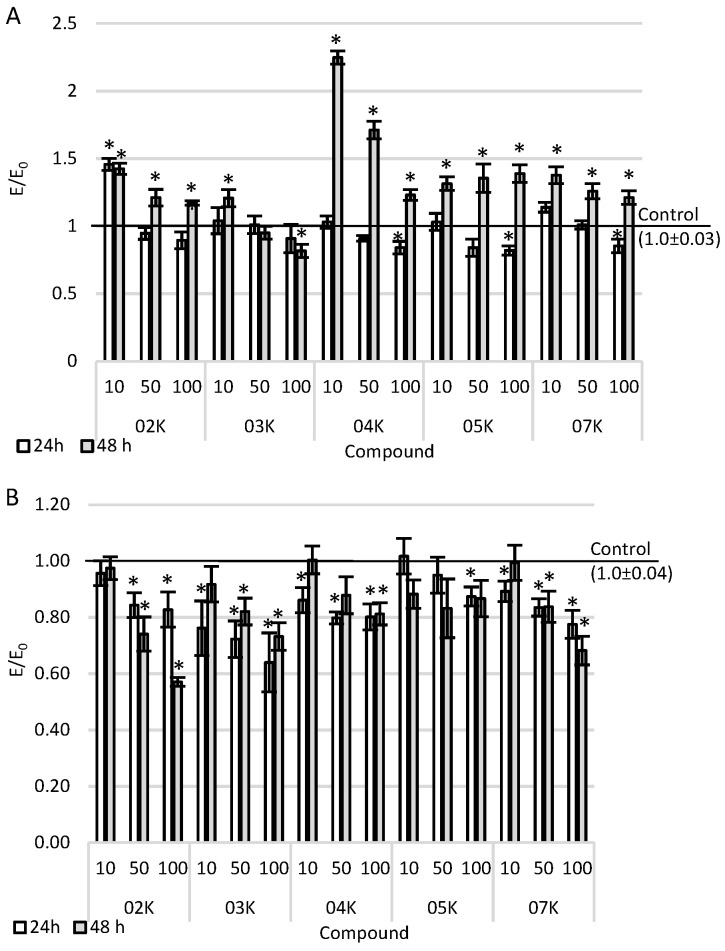
Cytotoxicity effect after the incubation of cells with the tested compounds: (**A**) NHDF cells; (**B**) A375 cells. Data are presented as means and SEMs (standard error of the mean). * *p* < 0.05—significant difference compared to the negative control.

The viability of NHDF and A375 cell cultures was assessed after 24-h and 48-h incubation periods with the tested compounds in the MTT test (Figure 1). They were expected to reduce the mitochondrial activity in tumor cells and not to affect normal cells. All the tested compounds showed dose-dependence—the higher the concentration was, the more cytotoxic the compound would be. The incubation of skin cancer cell cultures with O2K and O3K compounds resulted in a statistically significant reduction in the viability of the culture compared to the control (cell culture without the tested compounds only in the medium). At the same time, incubation with the O2K compound caused a reduction in activity of up to about 50% at a concentration of 100 µM after 48 h.

On the other hand, after the culturing of cells with O3K and the incubation period, a decrease in the cytotoxic effect of the tested compound was observed. The compound O4K also reduced the mitochondrial activity of skin cancer cell cultures by only about 20% at the highest concentration tested. The weakest effect was observed after incubating the cell culture with only 100 µM of O5K compound after 24 h of incubation. The decrease in viability was statistically significantly lower compared to that seen in the control. The compound O7K caused a time-dependent reduction in the mitochondrial activity of tumor cells in the concentration range of 50-100 µM: the longer the incubation was, the stronger the cytotoxic effect was. At the same time, the tested compounds showed a statistically significant cytotoxic effect after the 24-h incubation at the highest concentration tested with the compounds (except for the compound O2K). The tested compounds O4K, O5K, O7K, O2K, and O3K at a concentration of 10 µM after 48 h of incubation caused a statistically significant increase in mitochondrial activity in the whole range of tested concentrations compared to the control.

### 2.2. ROS and NO Scavenging

**Figure 2:**
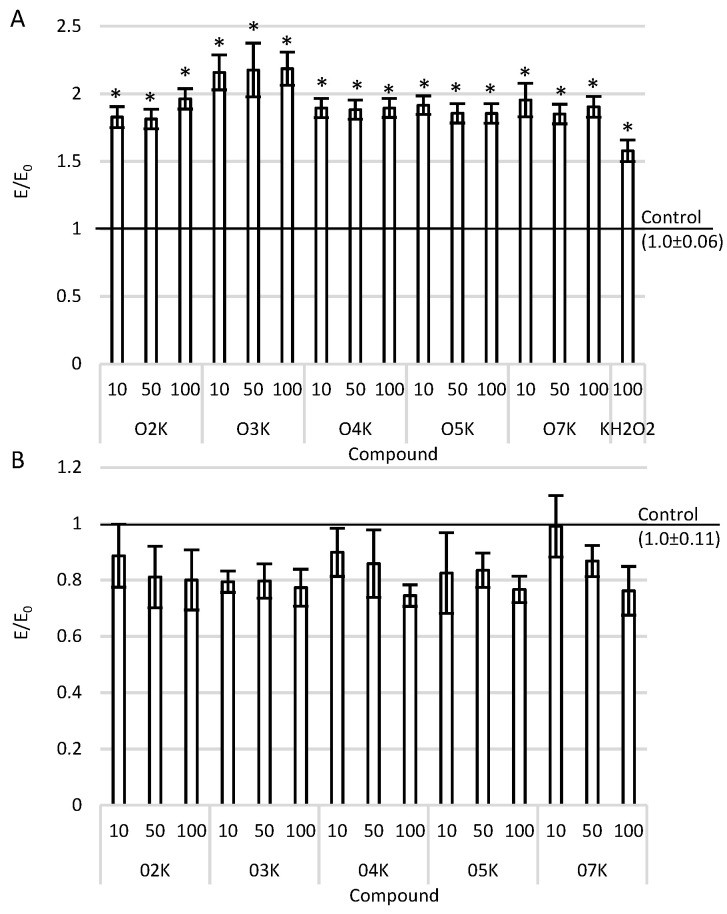
ROS (**A**) and NO (**B**) after incubation with the tested compounds in various concentrations in A375 cells. Data are presented as means and SEMs (standard error of the mean). * *p* < 0.05—significant difference than the negative control.

The influence of the tested compounds on the level of free oxygen radicals and nitric oxide was assessed (Figure 2). Anticancer compounds are expected to increase levels of free radicals, inducing ROS-mediated cancer-cell death and DNA strand damage. All the tested compounds in the whole range of tested concentrations caused an increase in free oxygen radicals compared to the negative control (cultured only in the medium) and the positive control (culture with 100 µM H_2_O_2_ for 1 h). However, none of the tested compounds caused an increase in nitric oxide. At the same time, as the concentration increased, a (statistically insignificant) decrease in the level of nitric oxide was observed compared to the negative control.

### 2.3. Glycoprotein-P Activity

**Figure 3:**
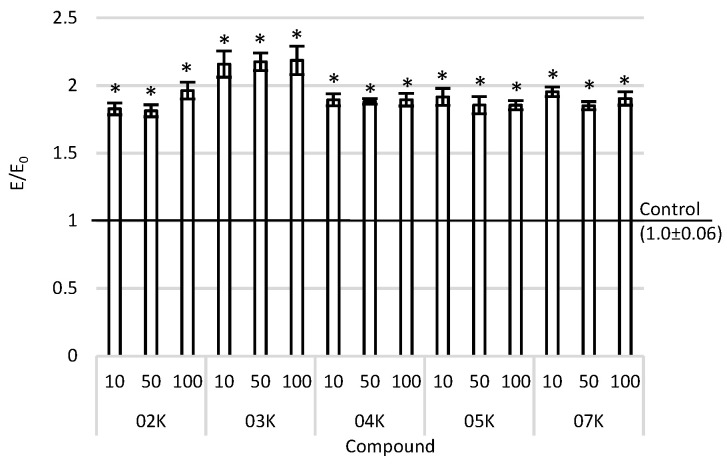
Rhodamine accumulation after incubation with the tested compounds in various concentration ranges in A375 cells. Data are presented as means and SEMs (standard error of the mean). * *p* < 0.05—significant difference than the negative control.

In normal tissues without changes, neoplasms, inflammations, and chronic exposure to increased levels of ROS may lead to changes in the initiation of oncogenesis. Along with the increased cell division, an intensive increase in the rate of metabolism can be observed. Hence, they show a higher level of free radicals than healthy tissues do. Despite their high ROS levels, tumors remain vulnerable to oxidative stress. The cytotoxicity of chemotherapeutic agents is often associated with ROS. However, the chronic presence of free radicals can cause drug resistance (P-gp, transmembrane proteins). The P-glycoprotein activity was tested by performing the Rh-123 accumulation assay. The accumulation of Rh-123 was observed in all tested compounds, regardless of the concentration used over the entire concentration range compared to the negative control (Figure 3). The highest accumulation of rhodamine was observed after the incubation of the skin cancer cells with the O3K compound.

### 2.4. Migration Assay

**Figure 4:**
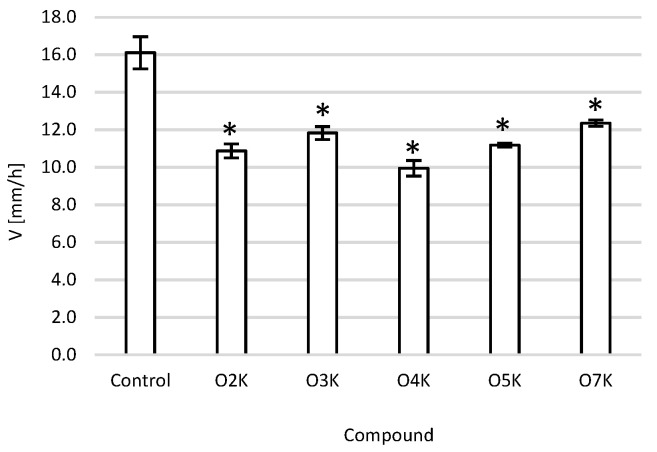
Effect of the tested compounds on the migration of cells in the scratch assay after 24 h of incubation for the A375 cell line: migration speed. * *p* < 0.05—significant difference than the negative control.

A scratch test was performed to assess tumor cell migration. The scratch width was measured after 24 h of incubation with the highest tested concentration of 100 µM of all the test compounds. The width after 24 h of incubation with the test compounds was subtracted from the width before incubation with the compounds and divided by 24 to determine the average space growth rate per hour. The fouling rate was expected to be slower after applying the test compounds compared to the negative control. All the tested compounds caused statistically significantly slower cell migration compared to the negative control. The strongest migration inhibition was observed in the presence of 100 µM of O4K and O2K compounds (Figure 4).

### 2.5. Detection of Apoptosis

**Figure 5:**
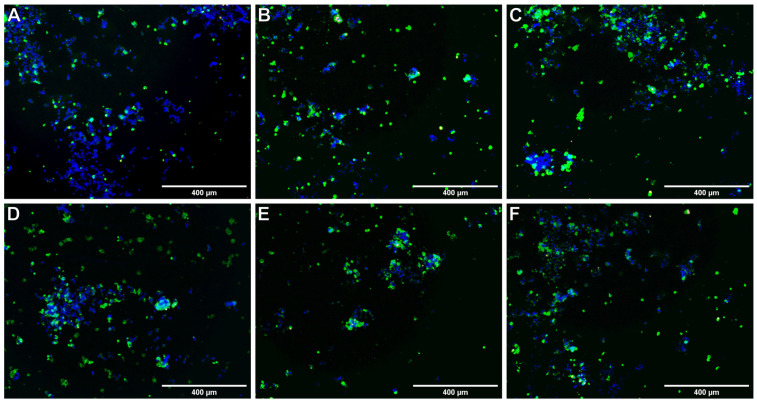
Apoptosis (green) and necrotic (red) cells after 24 h incubation with tested compounds at 10 μM (**A**), control; (**B**), O2K; (**C**), O3K; (**D**), O4K; (**E**), O5K; (**F**), O7K.

One of the goals of anticancer compounds is to increase the number of cells in the apoptotic phase (Figure 5). The concentrations of the tested compounds, which caused an increase in the frequency of apoptosis by 50% after 24 h, were calculated and are presented in Table 2. The obtained results confirmed the pro-apoptotic effect of all the tested compounds in A375 cell cultures. After 24 h of incubation, the highest pro-apoptotic effect was demonstrated by the compound O3K, and a slightly lower O2K. This effect in the case of the O3K and O4K compounds was slightly weaker than that in doxorubicin.

### 2.6. Multiple-Criteria Decision Analysis

**Figure 6:**
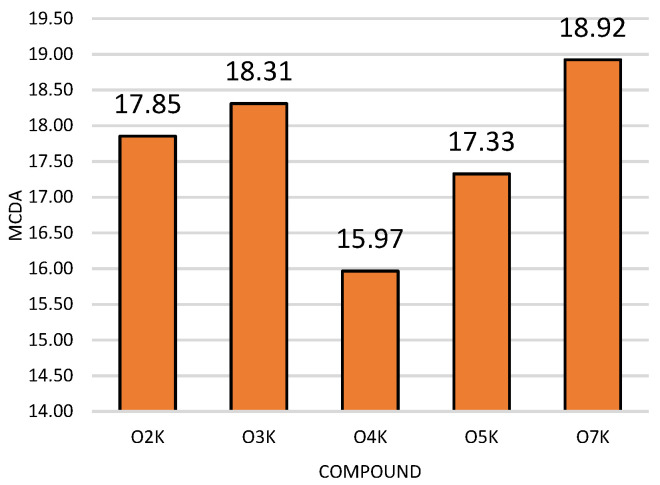
Multi-criteria decision analysis (MCDA) of the antitumor activity of the tested compounds.

The results obtained in the MTT, DCF-DA, Greiss, scratch, and apoptosis detection assays for the test compounds were analyzed using MCDA to compare the antitumor activity of the new compounds. The MCDA results (Figure 6) showed that compound O7K showed the strongest antitumor activity.

## 3. Discussion

Some compounds from this group of isoxazole derivatives have also been tested in murine cell lines [13]. The cytokine production by cells from the isolated lymphoid organs of 3 and 13-month-old mice and the possible mechanism of action of the investigated compounds in a model of Jurkat cells were determined [14]. Several in vivo models were used: humoral and cellular immune response, carrageenan inflammatory reaction, and the determination of lymphocyte subsets in non-immunized mice [15]. Within the last few years, immune evasion by cancer cells has become a popular and valuable therapeutic target to study [3].

This article includes an introduction to preclinical studies in new indications such as melanoma therapy, in which immunotherapy has successfully been used. We looked for more appropriate compounds in this group of derivatives in this type of cancer, and determined in this work whether the compounds have antitumor activity against melanoma. 

The compounds of the OK series (M series) are characterized by the immunomodulatory activity demonstrated in previous studies. Compound M2 containing a thiosemicarbazide group and an electronegative-chlorine atom in the 4-position of the phenyl ring, and M8 containing a semicarbazide and methoxyphenyl group, showed greater immunosuppressive properties than cyclosporine. In contrast, compound M7 showed the same immunosuppressive activity as cyclosporine [13]. In this study, selected immunomodulatory compounds were tested for their antitumor activity in relation to melanoma cancer.

The pharmacophore group, which is the isoxazole ring, is present in many of the compounds currently studied by other research teams in the field of antitumor activity. The series of novel isoxazole derivatives, morpholinyl propionamides, and piperazinyl propionamides were evaluated to determine their in vitro antitumor activity against HepG2, MCF-7, and HCT-116. Cell death was shown to occur mainly through apoptosis (supported by increased levels of caspases 3/9 and an increased Bax/Bcl-2 ratio) [16]. Moreover, 4,5,6,7-tetrahydro-isoxazole-[4,5-c]-pyridine derivatives showed significant antiproliferative and pro-apoptotic effects inducing both the early and late apoptosis of leukemic cell line K562 [17]. On the other hand, compounds containing 3,5-diarylisoxazole as the core of the structure showed antitumor activity against PC3 neoplastic cells of prostate cancer [18]. In addition, isoxazole-based chalcones and their dihydropyrazole derivatives bearing 2-fluoro-3,4-dimethoxyphenyl and 3,4-dimethoxyphenyl substituents showed excellent antitumor activity against the prostate cancer cell line (DU-145) [19].

Heterocycles improve the pharmacokinetic and pharmacodynamic properties of anticancer drugs by enhancing lipophilicity, polarity, or other physicochemical properties. Forskolin derivatives C1-isoxazole were tested using the breast cancer cell lines MCF-7 and BT-474 with a positive estrogen receptor. Most compounds were found to be active against p53-positive MCF-7 breast cancer cells, but not against p53-negative breast cancer cells BT-474 [20]. 

The isoxazole-piperazine derivatives had strong cytotoxicity on human liver cancer lines (Huh7 and Mahlavu) and breast cancer cell lines (MCF-7), with IC_50_ values ranging from 0.3 to 3.7 µM. The tested compounds inhibited the cell survival pathway by hyperphosphorylation and Akt apoptosis, as well as cell cycle arrest by activating the p53 protein [21]. The isoxazole carboxamide derivatives were also assessed for their cytotoxic activity on breast (MCF-7) and liver (Hep3B) cancer cell lines, as well as on the cervix (HeLa). Only two compounds from this series showed promising anticancer activity, delaying the G2/M phase by 18.07% [22].

The isoxazole [4,5-e] [1,2,4] triazepine derivatives were tested on six tumor cell lines. The best compound was 6-acetyl-8-phenyl-5-(propan-2-yl)-5,6-dihydro-4H [1,2] oxazolo [4,5-e] [1,2,4]-triazepine-3-carboxamide. At a dose of 10 μmol, it inhibited the growth of colon cancer cells (Colo-205, HCC-2988 and HT-29), CNS tumor cells (9F-296 and SF-539), melanoma cells (M-14, MDA-MB-435 and SK -ME), ovarian cancer cells (NCJ/ADR-RES), kidney cancer cells (RXF-393), prostate cancer cells (DV), and breast cancer cells (BT-549) [23].

The limitations of the study include the use of one melanoma line. In future studies, we plan to study more cell lines. However, there are several advantages to the A375 melanoma line in terms of designing experiments and then using potential test compounds in cancer therapy. During the characterization of melanoma cell lines, it was found that an increase in the acidic and basic isoforms of annexin-a1 (AnxA1) was found in A375 as compared to melanocytes and 526 cells. AnxA1 has been identified in mouse melanoma cell lines by comparative proteomic studies and appears to enhance melanoma invasion and spread. Moreover, it has recently been shown that tumor growth and metastasis are significantly decreased with the use of AnxA1-KO mice, suggesting the key role of AnxA1 in metastasis formation. The stress-response chaperone, PDI, was also expressed at a higher level in the A375 melanoma cell line. The PDI levels were reported to correlate with cancer invasion, metastasis, and drug resistance in other tumor types [24]. Moreover, VEGF receptors (VEGFR)1, VEGFR2, and neurophilin-1 are expressed in A375 melanoma cells. The forced overexpression of VEGF in these cells induces cell growth and triggers survival activity in serum-starved cultures by a mechanism dependent on the mitogen-activating protein-kinase signaling pathway [25]. Moreover, the A375 melanoma cell line contained the intrinsic endogenous fluorophore found in cell studies [26].

According to the act of 15 January 2015 on the protection of animals used for scientific or educational purposes in Poland and Directive of the European Parliament, and of the Council 2010/63/EU of 22 September 2010 on the protection of animals used for scientific purposes, the chosen method should allow for the most satisfactory results but cause as little pain, suffering, or distress as possible. The chosen method should minimize the number of animals used to produce reliable results and utilize species with the least susceptibility to pain, suffering, distress, and lasting harm, while being optimal for extrapolation to the target species. We selected compound O7K for further research and have applied for approval to conduct preclinical studies in mice. In this way, we will limit the number of animals on which the selected chemical compounds will have to be tested. 

## 4. Materials and Methods

### 4.1. Cell Line and Conditions

Normal human dermal fibroblast (NHDF) and human melanoma cell line (A375) were used in these studies. The first cell line was purchased from Lonza and the second from ATCC. Both cell lines were grown in 5% CO_2_ with 95% humidity at 37 °C. Twice a week, the morphology and confluence were evaluated under microscopy. If the confluence was above 70%, the cells were passaged with TrypLE (Gibco, Thermo Fisher Scientific, Waltham, MA, USA; cat. no. 12604-021) solution and used in a biological assay or 50% of the cells were removed. The NHDF cells were incubated in DMEM medium without phenol red (Lonza, Basel, Switzerland, cat. no. 12-604) and A431 in MEM medium (Lonza; cat. no. 12-611F). Both media were supplemented with fetal bovine serum (FBS; Biological Industries, Beit-Haemek, Israel, cat. no. 04-001-1A) to a final concentration of 10% and 2.0 mM L-glutamine (Lonza; cat. no. 17-605E) and antibiotics (penicillin and streptomycin). 

### 4.2. Viability Assay

The viability of cells was evaluated after using the tested compounds according to ISO 10993 part 5 Annex C–MTT assay. Both cell lines were seeded into 96-well plates at a density of 10,000 cells per well. The cells were incubated overnight to allow them to adhere to the surface of the well. The non-adherent cells were removed with supernatant and freshly prepared tested compounds were added for 24 or 48 h. After that time, the MTT solution was prepared at a 1 mg/mL concentration with PBS (Sigma-Aldrich, St. Louis, MO, USA, cat. no.: M2003-1G; Lonza; cat. no.: BE17-516F). The supernatant was removed and MTT solution was added for 2 h at 37 °C in 5% CO_2_ with 95% humidity. Then, the solution was gently removed and the violet crystal was dissolved in isopropanol (Pol-Aura, Olsztyn, Poland, cat.no.: 67-63-0) for 30 min by shaking. Finally, the absorbance was measured at 570 nm using a VariuScan microplate reader.

### 4.3. DCF-DA Assay and Greiss Assay

The antioxidant activity of tested compounds was tested in DCF-DA and Griess assay (cat. No. G7921; Thermo Fisher Scientific, Waltham, MA, USA), which evaluated reactive oxygen species (ROS) and nitric oxide (NO), respectively. After seeding both cells into 96-plates at a density of 30,000 cells, the culture plates were incubated at 37 °C in 5% CO_2_ with 95% humidity overnight. The next day, the supernatant was replaced with freshly prepared tested compounds and the cell culture was incubated at the same conditions for 1 h. Then, 50 µL of supernatant was transferred into the new plate. Next, the rest of the supernatant was removed and 25 µM of freshly prepared DCF-DA (Sigma-Aldrich; cat. no.: 35845) solution was added to the cells in PBS for 1 h at 37 °C in 5% CO_2_. A 50 µL mixture of reagent A and reagent B was added into the collected supernatant for 20 min in the dark at RT. The fluorescence was measured to evaluate the ROS level at ex. 485 nm and em. 535 nm and absorbance for NO level at 548 nm using a VariuScan microplate reader. 

### 4.4. Rh-123 Assay

To evaluate the glycoprotein-P activity, the Rh-123 assay was performed. First, the cells were seeded in the density of 30,000 cells per well and the next day the medium was replaced with freshly prepared tested compounds for 24 h. Then, 12.5 μM Rh-123 solution (Sigma-Aldrich; cat. no.: R8004-5MG) was added for 60 min. After that time, the culture plates were shaken at 500 g for 10 min and the supernatant was removed. Next, the cells were lysed in 20 mM Tris–HCl (pH 7.7) (Sigma-Aldrich; cat. no.: 10812846001) containing 0.2% sodium dodecyl sulfate (SDS; Pol-Aura; cat. no.: 151-21-3). Finally, the fluorescence was measured at ex. 485 nm/em538 nm using a VariuScan microplate reader.

### 4.5. Scratch Assay

A scratch test was performed to assess whether the test compounds could inhibit skin cancer metastasis and infiltration. After seeding the cells, they were incubated until the cells were in a monolayer on the whole surface of the well. Then, the scratch test was performed, and the freshly prepared, tested compounds at 100 µM were added. Next, the microphotographs were taken using EVOS FL microscopy and the culture plates were incubated for 24 h. The next day, the microphotographs were again taken. Finally, the width of the scratch was measured using ImageJ open-space platform. 

### 4.6. Detection of Apoptosis 

The A375 cells were grown in 96-well plates for 24 h to allow the cells to adhere to the surface of the wells. Compounds were tested in a concentration range of 10–100 µM after 24 h of incubation with the cells. After this time, the medium was changed to an Annexin-V mixture conjugated with fluorescein and propidium iodide in PBS with Mg^2+^ and Ca^2+^ ions (Thermo Fisher Scientific, Waltham, MA, USA; cat. no. V13242). The plate was incubated for 20 min at 37 °C, then pictures were taken under a fluorescence microscope. Thirteen images were taken from each well using an EVOS FL fluorescence microscope. Each compound was performed in three replications. Then, the number of cells was analyzed using the open ImageJ space.

### 4.7. Statistical Analysis 

All results are presented as means ± SEMs. Biological assays were performed in five replicates, and each replicate used five samples. The data had a normal distribution and equality of variance, so a one-way ANOVA with post-hoc Tukey’s was performed. The control was the cell culture incubated only with the appropriate medium without the tested compounds. In the DCF-DA assay, a second control was used: a positive control (cells incubated with 100 µM H2O2 for 1 h). The statistical analyses were carried out in the STATISTICA v 13 program. The p-value was set at 0.05. In order to select the most promising compound for further research, multi-criteria decision analysis (MCDA) was performed using the weighted sum model (WSM). 

All results are presented as the mean ± SEM (standard error of the mean) relative to the respective control (E/E_0_), where E is the result for the measured sample and E_0_ is the result for the negative control.

## 5. Conclusions

It was shown in the conducted studies that all the tested compounds had antiproliferative activity towards melanoma cell lines and the strongest, O4K and O2K compounds, inhibited the formation of metastases, as evidenced by the slower migration of tumor cells after the administration of the test compounds. Moreover, the mechanism of the antitumor activity of the tested compounds was confirmed—i.e., the pro-apoptotic activity of the compounds in A375 cell cultures. The compound O3K showed the highest pro-apoptotic effect.

The accumulation of Rh-123, regardless of the concentration used, was observed in all tested compounds over the entire concentration range compared to the negative control, indicating their ability to inhibit P-glycoprotein, which is the major transporter involved in multidrug resistance in humans. The information obtained brings us closer to a more rational search for compounds capable of a pharmacological reduction in cancer cell resistance to chemotherapy.

The MCDA results showed that O7K showed the strongest antitumor activity. The compound O7K contains a methoxyphenyl substituent with electron-donating properties and at the same time is the compound with the lowest hydrophobicity out of all the examined structures. Compound O7K has been marker for further stages of research on potential compounds to be used in therapy supporting oncological chemotherapy.

Chemotherapy is widely used, especially against inoperable tumors, as a basic therapy or as an adjunct treatment before and/or after subsequent treatment. However, the use of chemotherapy is limited because it has a poor efficacy; minimal target cell selectivity; and undesirable side effects, such as alopecia, nausea, and vomiting [22]. It is very important to search for new components of oncological therapy that will ensure the greater effectiveness of the therapy and, at the same time, will be free from troublesome side effects reducing the quality of life of an oncological patient.

## Figures and Tables

**Table 1:** Calculated concentrations of test compounds that increase the rate of apoptosis by 50% in A375 cells after 24 h.

Compounds	Structure
O2K	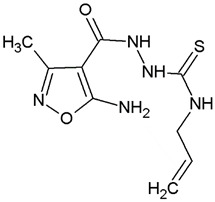
O3K	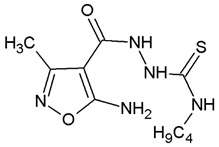
O4K	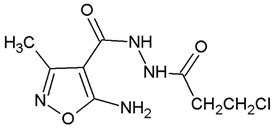
O5K	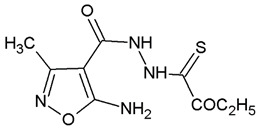
O7K	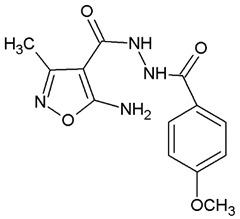

**Table 2:** Calculated concentrations of test compounds which increased the rate of apoptosis by 50% in A375 cells after 24 h.

Compounds	AC_50_ (SD) [µM]
O2K	4.861 (0.187)
O3K	3.517 (0.359)
O4K	3.852 (0.278)
O5K	5.277 (0.301)
O7K	5.547 (0.167)
Doxorubicin	3.189 (0.421)

## Data Availability

The data will be made available after contact with the author of the correspondence.

## References

[1] Rahmati M., Ebrahim S., Hashemi S., Motamedi M., Moosavi M.A. (2020). New insights on the role of autophagy in the pathogenesis and treatment of melanoma. Mol. Biol. Rep..

[2] Hessler M., Jalilian E., Xu Q., Reddy S., Horton L., Elkin K., Manwar R., Tsoukas M., Mehregan D., Avanaki K. (2020). Melanoma biomarkers and their potential application for in vivo diagnostic imaging modalities. Int. J. Mol. Sci..

[3] Eddy K., Chen S. (2020). Overcoming immune evasion in melanoma. Int. J. Mol. Sci..

[4] Quaresmini D., Guida M. (2020). Neoangiogenesis in Melanoma: An Issue in Biology and Systemic Treatment. Front. Immunol..

[5] Weidle U.H., Auslander S., Brinkmann U. (2020). Micro RNAs promoting growth and metastasis in preclinical in vivo models of subcutaneous melanoma. Cancer Genom. Proteom..

[6] Simiczyjew A., Dratkiewicz E., Mazurkiewicz J., Ziętek M., Matkowski R., Nowak D. (2020). The influence of tumor microenvironment on immune escape of melanoma. Int. J. Mol. Sci..

[7] Kato J., Uhara H. (2021). Immunotherapy for advanced melanoma: Current situation in Japan. Jpn. J. Clin. Oncol..

[8] Olaoba O.T., Kadasah S., Vetter S.W., Leclerc E. (2020). Rage signaling in melanoma tumors. Int. J. Mol. Sci..

[9] Sysak A., Obmińska-Mrukowicz B. (2017). Isoxazole ring as a useful scaffold in a search for new therapeutic agents. Eur. J. Med. Chem..

[10] Zimecki M., Bachor U., Maczyński M. (2018). Isoxazole derivatives as regulators of immune functions. Molecules.

[11] Pang G.X., Niu C., Mamat N., Aisa H.A. (2017). Synthesis and in vitro biological evaluation of novel coumarin derivatives containing isoxazole moieties on melanin synthesis in B16 cells and inhibition on bacteria. Bioorg. Med. Chem. Lett..

[12] Yin L., Niu C., Liao L.X., Dou J., Habasi M., Aisa H.A. (2017). An isoxazole chalcone derivative enhances melanogenesis in b16 melanoma cells via the Akt/GSK3β/β-catenin signaling pathways. Molecules.

[13] Mączyński M., Zimecki M., Taraszkiewicz M., Ryng S. (2008). Synthesis, immunological activity and computional study of 5-amino-3-methyl-4-izoxazolecarboxylic acid semicarbazides and thiosemicarbazides. Acta Pol. Pharm. Drug Res..

[14] Drynda A., Obmińska-Mrukowicz B., Zaczyńska E., Zimecki M., Kochanowska I., Ryng S., Mączyński M. (2017). 5-Amino-3-methyl-4-isoxazolecarboxylic acid hydrazide derivatives with in vitro immunomodulatory activities. Chem. Biol. Drug Des..

[15] Drynda A., Obmińska-Mrukowicz B., Zaczyńska E., Zimecki M., Ryng S., Mączyński M. (2016). Immunoregulatory effects of 4-(4-chlorophenyl)-1-(5-amino-3-methylisoxazole-4-carbonyl)-thiosemicarbazide (06K) in non-immunized and SRBC-immunized mice. J. Pharm. Pharmacol..

[16] Warda E.T., Shehata I.A., El-Ashmawy M.B., El-Gohary N.S. (2020). New series of isoxazole derivatives targeting EGFR-TK: Synthesis, molecular modeling and antitumor evaluation. Bioorg. Med. Chem..

[17] Lampronti I., Simoni D., Rondanin R., Baruchello R., Scapoli C., Finotti A., Borgatti M., Tupini C., Gambari R. (2020). Pro-apoptotic activity of novel synthetic isoxazole derivatives exhibiting inhibitory activity against tumor cell growth in vitro. Oncol. Lett..

[18] Aktaş D.A., Akinalp G., Sanli F., Yucel M.A., Gambacorta N., Nicolotti O., Karatas O.F., Algul O., Burmaoglu S. (2020). Design, synthesis and biological evaluation of 3,5-diaryl isoxazole derivatives as potential anticancer agents. Bioorg. Med. Chem. Lett..

[19] Shaik A., Bhandare R.R., Palleapati K., Nissankararao S., Kancharlapalli V., Shaik S. (2020). Antimicrobial, antioxidant, and anticancer activities of some novel isoxazole ring containing chalcone and dihydropyrazole derivatives. Molecules.

[20] Burra S., Voora V., Rao C.P., Vijay Kumar P., Kancha R.K., Krupadanam G.L.D. (2017). Synthesis of novel forskolin isoxazole derivatives with potent anti-cancer activity against breast cancer cell lines. Bioorg. Med. Chem. Lett..

[21] Çalışkan B., Sinoplu E., İbiş K., Güzelcan E.A., Atalay R.Ç., Banoglu E. (2018). Synthesis and cellular bioactivities of novel isoxazole derivatives incorporating an arylpiperazine moiety as anticancer agents. J. Enzym Inhib. Med. Chem..

[22] Eid A.M., Hawash M., Amer J., Jarrar A., Qadri S., Alnimer I., Sharaf A., Zalmoot R., Hammoudie O., Hameedi S. (2021). Synthesis and Biological Evaluation of Novel Isoxazole-Amide Analogues as Anticancer and Antioxidant Agents. Biomed. Res. Int..

[23] Wagner E., Wietrzyk J., Psurski M., Becan L., Turlej E. (2021). Synthesis and Anticancer Evaluation of Novel Derivatives of Isoxazolo[4,5-e][1,2,4]triazepine Derivatives and Potential Inhibitors of Protein Kinase C. ACS Omega.

[24] Caputo E., Maiorana L., Vasta V., Pezzino F.M., Sunkara S., Wynne K., Elia G., Marincola F.M., McCubrey J.A., Libra M. (2011). Characterization of human melanoma cell lines and melanocytes by proteome analysis. Cell Cycle.

[25] Graells J., Vinyals A., Figueras A., Llorens A., Moreno A., Marcoval J., Gonzalez F.J., Fabra A. (2004). Overproduction of VEGF165 concomitantly expressed with its receptors promotes growth and survival of melanoma cells through MAPK and PI3K signaling. J. Investig. Dermatol..

[26] Shirkavand A., Mohajerani E., Farivar S., Ataie-Fashtami L., Ghazimoradi M.H. (2021). Quantitative Autofluorescence Imaging of A375 Human Melanoma Cell Samples: A Pilot Study. J. Lasers Med. Sci..

